# Endemic Persistence of Bovine Leukemia Virus in aHigh‐Producing Brazilian Dairy Herd: Seroprevalence Across Production Strata, Vertical Transmission, and Mortality Risk

**DOI:** 10.1155/vmi/5252185

**Published:** 2026-09-04

**Authors:** Karla Cristina Resplandes da Costa Paz, Leonardo Costa Paes, Dirceu Guilherme de Souza Ramos, Rosângela Maria Rodrigues, Eduardo Vignoto Fernandes, João Batista Alves de Souza, Adeliane Castro da Costa, Ísis Assis Braga, Cecília Nunes Moreira

**Affiliations:** ^1^ Graduate Program in Bioscience and One Health, Institute of Agricultural Sciences, Federal University of Jataí, Jataí, Goiás, Brazil; ^2^ Graduate Program in Applied Health Sciences, Institute of Biosciences, Federal University of Jataí, Jataí, Goiás, Brazil, unesp.br

**Keywords:** BLV, enzootic bovine leukosis, lymphocytosis, multiparous cows, vertical transmission

## Abstract

Bovine leukemia virus (BLV) is globally distributed and remains difficult to control. The infection is associated with substantial economic losses resulting from reduced productivity and early culling. Seroepidemiological studies conducted in Brazil have demonstrated that BLV prevalence rates range from 18.30% to 46.76%, with prevalence estimates reaching up to 90% in Goiás State, where the present study was performed. This study investigates the silent epidemiological persistence of BLV in a high‐producing dairy herd, highlighting category‐specific seroprevalence patterns, serological evidence suggestive of possible vertical exposure, and the association between BLV seropositivity and mortality. A total of 570 Girolando females were divided into the following groups for analysis of anti‐BLV antibodies: multiparous (I), primiparous (II), bred nulliparous heifers (III), and nonbred heifers (IV). We performed the analysis using a commercial enzyme‐linked immunosorbent assay. Our research group evaluated the impact of BLV seropositivity on milk yield (MY), somatic cell count (SCC), services per conception (SC), and calving interval (CI). In addition, serological evidence suggestive of possible vertical exposure and herd incidence rates were assessed during a 3‐month follow‐up period. Prevalence of BLV seropositivity was 59.82% (95% CI: 55.75%–63.77%). Cows in Group I showed a significantly higher likelihood of seropositivity than those in Group IV, with an approximately 8.6‐fold (95% CI: 4.96–15.03) increased risk of BLV seropositivity. Serological findings suggestive of possible vertical exposure were detected in 13.59% of the evaluated neonates, and the BLV incidence rate in the herd was 34.06 new seropositive cases over the 3‐month follow‐up period. Despite the high seroprevalence observed in the herd, no adverse effects were associated with productive or reproductive parameters. No significant association was observed between BLV seropositivity and lymphocytosis (*p* > 0.05). In contrast, mortality was significantly higher among BLV‐seropositive cattle than among seronegative cattle (RR = 1.41; 95% CI: 1.19–1.67). The impact of this disease on the lifespan of the herd should not be underestimated, particularly when considering mortality rates, incidence, and vertical transmission, which underscore the progressive nature of the virus.

## 1. Introduction

Bovine leukemia virus (BLV), the etiological agent of enzootic bovine leukosis, is widespread in ruminants worldwide. The estimated global prevalence is approximately 19%, with higher rates in North America, followed by Asia, Africa, South America, and Europe [1, 2]. In South America, BLV is considered endemic, with reported prevalence ranging from 27% to 77%. In Brazil, seroprevalence exceeds 50%, although marked regional variations have been described, including 40.13% in the Midwest, 34.41% in the South, 46.72% in the Southeast, 29.94% in the Northeast, and approximately 18.30% in the North [3–6].

Transmission occurs primarily through horizontal spread, either by direct contact or through iatrogenic practices involving contaminated fluids. Sources of infection include carrier animals that shed the virus in blood, semen, saliva, colostrum, milk, and nasal secretions containing infected lymphocytes [7–9]. Furthermore, vertical transmission during the perinatal period has been reported in 4%–10% of cases [10].

This infection is characterized by a prolonged incubation period ranging from 1 to 5 years, with detectable seroconversion occurring within 2–3 weeks after exposure. As a chronic and subclinical infection, approximately 70% of infected cattle remain asymptomatic, and only about 10% develop persistent lymphocytosis or lymphosarcoma, leading to mortality and premature culling. In advanced stages, clinical signs may include digestive disorders, anorexia, and weight loss [11, 12].

Although BLV infection is frequently subclinical, it has been associated with negative impacts on animal longevity, including qualitative and quantitative milk losses and impaired reproductive performance [13, 14]. Moreover, the infection may result in restrictions on international trade, reduced productive lifespan associated with immunosuppression, increased susceptibility to secondary infections, carcass condemnation, and elevated sanitary and economic costs [6]. Given the lack of effective vaccines or treatments, prevention relies primarily on the segregation of infected animals and proper sanitary management practices [11].

BLV genetic material has been detected in human tissues, and some studies have suggested a possible association with breast cancer. However, current evidence remains inconclusive regarding its zoonotic potential. In addition, milk has been proposed as a potential source of exposure, as proviral sequences have been identified in this secretion and may be capable of infecting susceptible cells, although no definitive transmission pathway has been established [15–18].

This study aimed to evaluate the productive and reproductive effects of infection across different animal strata within a herd. We also examined the prevalence of vertical transmission and the hematological alterations in clinically healthy but seropositive animals. These aspects are critical for the implementation of effective control measures, and serological screening of the herd is recommended to identify infected animals, including those in subclinical stages [19].

## 2. Materials and Methods

### 2.1. Study Area and Sample Characteristics

The study was conducted on a Free Stall dairy farm with advanced technology. It has an approximate production of 8000 liters (L) of milk/day. The farm is in the municipality of Jataí, in Goiás State. All cows in the herd were included in the analysis between June 2023 and June 2024, ensuring complete population coverage.

Anti‐BLV antibody detection was performed in 570 crossbred female cattle (Girolando), including 315 multiparous cows (Group I), 113 primiparous cows (Group II), 33 bred nulliparous heifers (Group III), and 109 nonbred heifers (Group IV) that had not undergone reproductive management.

During the research, 333 cows were lactating, making an average of 30.1 liters of milk per animal/day. They had productive data collected throughout the experimental period, such as milk yield (MY) and somatic cell count (SCC) means. We verified the reproductive data by analyzing the number of services per conception (SC) and calving interval (CI) in 436 animals belonging to Groups I, II, and III, while the number of CI was evaluated in 204 cows of Groups I and II. During the study period, 103 neonates born to BLV‐seropositive cows were evaluated for serological evidence suggestive of possible vertical exposure.

### 2.2. Sample Collection and Processing

After individual restraint, 10 mL of whole blood was collected from the coccygeal vein of females in Groups I, II, III, and IV. Also, 5 mL of whole blood from the jugular vein of neonates of seropositive cows was collected prior to colostrum ingestion, supplied through a colostrum bank. After antisepsis, the sample was collected in a vacutainer system in tubes without anticoagulant to obtain serum. They were stored in microtubes and kept at a temperature of −20°C until the samples were processed.

The commercial enzyme‐linked immunosorbent assay kit (Leukosis Blocking Ab Test, IDEXX Laboratories, Inc., Westbrook, ME, EUA) was used to detect the antibodies against BLV gp51 antigen, following the manufacturer’s guidelines.

Hematological data, including erythrogram, leukogram, platelet count, and fibrinogen, were evaluated in 65 BLV‐seropositive cows and 38 BLV‐seronegative cows, using reference values from Kerr [20]. The 103 cows were randomly selected at 30‐day intervals over three months (T1, T2, and T3).

### 2.3. Analysis of Risk Factors and Productive Impacts

The frequency of seropositive cows and calves was calculated using a 95% confidence interval [21]. The following parameters were evaluated to check the effects of BLV seropositivity on the productive and reproductive indices of the animals over a one‐year period: (i) Reproductive category–Groups‐I, II, III, and IV; (ii) cattle mortality (not including cows that died due to trauma, poisoning, metabolic ketosis, parasitic sadness, snakebite, tetanus, and animals discarded for consumption); (iii) MY (≤ 19.9 L/day and ≥ 20 L/day); (iv) SCC (≤ 500 thousand cells/mL and ≥ of 501 thousand cells/mL); (v) CI (≤ 365 days and ≥ 366 days); and (vi) SC (≤ 3 and ≥ than 4). Associations between variables were assessed using the chi‐square test, considering *p* ≤ 0.05 as statistically significant. When expected frequencies were low, Fisher’s exact test was applied. Odds ratios (ORs) and relative risks (RRs) were calculated for variables associated with BLV seropositivity. Because the primary objective of this study was to provide a descriptive epidemiological assessment of BLV infection in a single dairy herd, the statistical analysis focused on univariable methods. All statistical analyses were performed using Epi Info Version 7.2.

Hematological parameters were evaluated and compared between BLV‐seropositive and BLV‐seronegative cows over the study period. The data at different evaluated times were compared using the nonparametric Mann–Whitney test, considering a minimum significance index of *p* ≤ 0.05. We used GraphPad Prism 6.0 software for statistical analysis.

The incidence rate was assessed three months after the first test, an interval defined to allow seroconversion following BLV, including 229 seronegative animals and calculated as the number of new cases divided by the population at risk.

### 2.4. Ethics Committee

The Ethics Committee on the Use of Animals of the Federal University of Jataí (CEUA‐UFJ) approved this research under the protocol 004/2023.

## 3. Results

The prevalence of BLV‐seropositive animals on the farm was 59.82% (95% CI: 55.75%–63.77%). The presence of anti‐BLV antibodies was significantly associated with reproductive category. Multiparous cows exhibited a markedly higher likelihood of seropositivity compared to nonbred heifers (OR = 61.25; 95% CI: 28.99–129.4). The corresponding RR indicated an approximately 8.6‐fold increase (95% CI: 4.96–15.03) in the risk of BLV seropositivity in multiparous cows (Table 1). Mortality reached 82.35% among BLV‐seropositive animals, compared to 58.40% in seronegative cattle (Fisher’s exact test, *p* = 0.006). BLV‐seropositive animals had a 41% higher risk of death than seronegative cattle (RR = 1.41; 95% CI: 1.19–1.67), and the odds of death were 3.32 times higher (OR = 3.32; 95% CI: 1.35–8.16).

**TABLE 1:** Frequency of cows with anti‐bovine leukemia virus antibodies, represented by reproductive category from a dairy farm with advanced technology.

Reproductive category		*n* = 570	Seropositive cow		*p* value
			*n*	% (95% CI)	
Group	I	315	275	87.30 (83.17–90.53)	0.001
	II	113	50	44.25 (34.91–53.89)	
	III	33	5	15.15 (5.11–31.90)	
	IV	109	11	10.09 (5.15–17.34)	

*Note:* I, multiparous cows; II, primiparous cows; III, nulliparous cows submitted to reproductive management; IV, heifers that have not undergone reproductive management; 95% CI, confidence interval.

Although 94.29% (95% CI: 91.26–96.32) of the 333 lactating dairy cows were seropositive, BLV seropositivity was not associated with production parameters, including MY and SCC. Likewise, no effect was observed on the reproductive performance of 204 cows, as measured by the number of SC. However, the 436 seropositive cows from Groups I, II, and III were more likely to present a CI ≤ 365 days. Nevertheless, the RR estimate was imprecise and not statistically significant (RR = 2.48; 95% CI: 0.88–7.03) (Table 2).

**TABLE 2:** Evaluation of the effect of bovine leukemia virus seropositivity on productive and reproductive indicators of crossbred cows from a dairy farm with advanced technology.

Indicators	Category	Seropositive cow		*p* value
		*n*	% (95% CI)	
MY	≤ 19.9	62/314	19.75 (15.72–24.50)	1.000
	≥ 20	252/314	80.25 (75.50–84.28)	

SCC	≤ 500,000	242/314	77.07 (72.11–81.38)	0.266
	≥ 501,000	72/314	22.93 (18.62–27.89)	

SC	≤ 3	234/378	61.90 (56.91–66.66)	0.469
	≥ 4	144/378	38.10 (33.34–43.09)	

CI	≤ 365	82/187	43.85 (36.62–51.28)	0.041^∗^
	≥ 366	105/187	56.15 (48.72–63.38)	

*Note:* MY, milk yield (L/day); SCC, somatic cell count (cells/mL); CI, calving interval (days); 95% CI, confidence interval.

Abbreviation: SC, services per conception.

^∗^(OR = 3.64; 95% CI: 1.01–13.11).

Anti‐BLV antibody detection analysis in neonates of seropositive cows indicated 13.59% seroreactivity (95% CI: 7.63–21.75). A total of 78 cows became seropositive during the three‐month follow‐up, resulting in an incidence rate of 34.06 new cases per 100 animals at risk.

Regarding the hematological analysis of the seronegative and seropositive groups, the average red blood cell count was 5.86 ± 0.13 and 5.72 ± 0.98 (10^6^/mm^3^), platelets 238 ± 21.34 and 212.80 ± 57.05 (10^3^/μL), and fibrinogen 446.33 ± 10.02 and 413.00 ± 43.55 (mg/dL), respectively, with no difference between the averages of cows exposed and not exposed to BLV. The leukogram results according to the seropositivity status of the animals over time are presented in Table 3.

**TABLE 3:** Leukocyte counts in 103 dairy cows according to BLV serological status (65 seropositive and 38 seronegative) evaluated at three time points with 30‐day intervals.

	T1		T2		T3	
	Seropositive	Seronegative	Seropositive	Seronegative	Seropositive	Seronegative
Total leukocytes	11,089 ± 5287	10,728 ± 3689	12,824 ± 7957	12,021 ± 4909	11,575 ± 6687	9920 ± 3517
Monocytes	453 ± 404	444 ± 336	553 ± 442	556 ± 419	421 ± 250	439 ± 289
Lymphocytes	10,327 ± 9391^∗^	7397 ± 6778	8271 ± 6417^∗^	6849 ± 2958	7520 ± 5762^∗^	5588 ± 2687
Eosinophils	373 ± 261	476 ± 512	451 ± 439	377 ± 338	467 ± 597	291 ± 384
Total neutrophils	3787 ± 2266	3888 ± 1942	3630 ± 2189	4525 ± 3217	3710 ± 1959	4058 ± 2661
Bands neutrophils	255 ± 315^∗^	397 ± 482^∗^	347 ± 389^∗^	252 ± 165^∗^	310 ± 746^∗^	203 ± 181^∗^
Segmented neutrophils	3495 ± 1874	3761 ± 1676	3260 ± 2049	4333 ± 3130	3296 ± 2086	3903 ± 2632

*Note:* Data are presented as mean ± standard deviation. Leukocytes (cell/μL); T1, Time 1; T2, Time 2; T3, Time 3.

^∗^Cell counts above the species reference interval.

**FIGURE 1:**
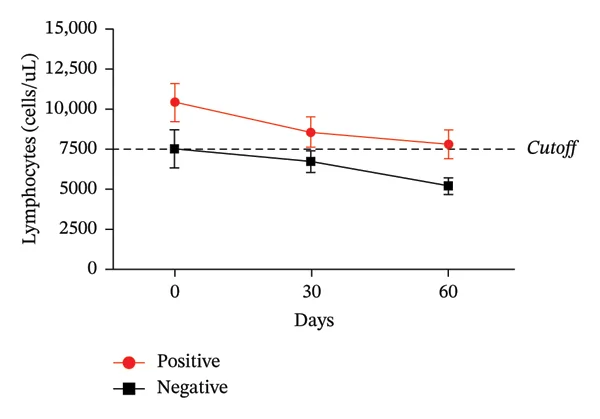
Lymphocyte counts (cells/μL) in 103 cows tested for antibodies against bovine leukemia virus (65 seropositive and 38 seronegative) from a high‐producing dairy herd. Blood samples were collected at 30‐day intervals over a 3‐month follow‐up period (Days 0, 30, and 60). The dashed line represents the cutoff value for lymphocytosis. Lymphocyte counts among seropositive cows were compared across the three sampling time points using one‐way ANOVA followed by Bonferroni’s multiple comparisons test. No significant differences were observed (*p* > 0.05).

Slightly increased band neutrophil counts were observed in cows from both groups at all three evaluation times (*p* > 0.05), whereas seropositive cows exhibited lymphocytosis throughout the study period. Although the normal parameter of lymphocyte count in BLV seronegative cows was observed, there was a difference between the means of the seropositive and seronegative groups. It was not statistically significant, and no differences were observed between the means at the different time points evaluated (Figure 1).

## 4. Discussion

The high BLV seroprevalence observed in this herd indicates that the infection remains widely distributed even in technologically advanced dairy production systems. This finding is consistent with reports from Brazil and other endemic regions, where prevalence may reach up to 60%, although it varies according to management practices and herd characteristics [1, 3–7] and indicates that technological advancement alone is insufficient to control viral spread, reinforcing the role of management‐related factors [11, 22].

In this context, the association between multiparous cows and seropositivity observed in this study is consistent with previous findings, indicating that cumulative exposure to management practices, such as rectal palpation, injectable treatments, and milking procedures, represents a major factor in BLV transmission [23], reinforcing the relevance of horizontal transmission as the primary route of infection in adult animals.

However, the detection of anti‐BLV antibodies in calves and heifers indicates that younger categories also contribute to the maintenance of viral circulation within the herd. Despite the use of pasteurized colostrum and the absence of direct contact between cows and calves, serological findings suggestive of possible vertical exposure were observed in a proportion of the evaluated neonates. These findings are consistent with previous reports indicating that vertical transmission of BLV may occur under field conditions, although it cannot be confirmed based solely on serological evidence [24]. Gutiérrez et al. [25] demonstrated that animals infected early in life may develop increasing proviral loads over time; however, this biological mechanism was not evaluated in the present study. Therefore, our findings suggest that postnatal management practices alone may not fully explain the occurrence of BLV‐seropositive neonates and warrant further investigation using molecular diagnostic approaches.

The absence of significant effects of BLV seropositivity on MY and scc is consistent with previous studies [13, 14]. However, although BLV seropositivity alone is not associated with productive losses, the literature indicates that proviral load represents a more sensitive parameter for detecting productive impacts, particularly in animals with high viral loads [8, 13].

Similarly, no significant effects of BLV infection were observed on reproductive parameters, particularly SC and CI. This finding corroborates studies based on serological diagnosis [26]. However, in endemic regions, animals with high proviral load may exhibit reproductive impairments, including prolonged calving‐to‐conception intervals and increased susceptibility to subclinical diseases such as mastitis and ketosis [1, 14].

From a hematological perspective, there were no significant differences observed in erythrocyte, leukocyte, platelet, or fibrinogen counts between seropositive and seronegative groups. Moreover, there was no association between infection with BLV and changes in these parameters over time. Ruiz et al. [27] reported a similar result using serological methods, but they found higher total leukocyte counts in cattle with high viral load, analyzed by quantitative PCR. A study conducted in Pakistan on purebred Holstein–Friesland cattle reported important hematobiochemical effects, such as increased red and white cell counts, in seropositive animals [28].

Although lymphocytosis was observed in cows seropositive for BLV, it was not possible to confirm the occurrence of persistent lymphocytosis, as the mean lymphocyte count decreased over time. Persistent lymphocytosis is considered an important risk factor for BLV transmission and occurs in approximately 30% of infected cattle, particularly in those with a high load of infected cells in peripheral blood, and this process is associated with increased proliferation of B lymphocytes and a reduction in CD4+ T lymphocytes, driven by decreased apoptosis of CD5+ cells [29]. On the other hand, the increased proportion of immature neutrophils in peripheral blood reflects a bone marrow response to inflammatory and infectious processes, particularly of bacterial origin [30].

The pathogenesis of BLV infection may vary depending on viral and host‐related factors, with differences in virulence among BLV genotypes representing an important determinant of disease progression [11]. However, viral genotyping was not performed in the present study, and thus the potential influence of viral genetic variability on the observed results cannot be inferred.

Mortality was significantly associated with BLV seropositivity, with seropositive cattle showing a 41% higher mortality risk than seronegative cattle. However, no necropsy, histopathological examination, or molecular confirmation was performed in animals that died; therefore, these findings should be interpreted as an epidemiological association rather than evidence of a causal relationship. To minimize potential bias, animals with confirmed alternative causes of death were excluded from the analysis. Previous studies have reported reduced longevity, increased mortality, and earlier culling among BLV‐seropositive cattle, resulting in substantial productive and economic losses [23].

In addition, approximately 35 new seroconversion cases were identified during the three‐month follow‐up period, suggesting continued BLV circulation within the herd in the absence of overt clinical disease. However, because only serological testing was performed, it was not possible to determine whether these seroconversions reflected delayed serological responses to previous exposure or newly acquired infections.

## 5. Conclusions

The high seroprevalence observed in this study demonstrates sustained BLV circulation within a high‐producing dairy herd despite the absence of overt clinical disease. Higher seropositivity was identified among adult animals, suggesting cumulative exposure over time, while serological findings in a proportion of neonates born to seropositive cows were suggestive of possible vertical exposure, warranting further investigation using molecular methods. The association between BLV seropositivity and increased mortality highlights the need for continued surveillance, although the contribution of BLV infection to the observed mortality could not be definitively established in the present study.

These findings reinforce the importance of routine serological screening, particularly in clinically healthy herds, as an essential tool for identifying infected animals and monitoring BLV circulation in endemic dairy systems. However, serological testing alone cannot fully characterize infection dynamics or transmission patterns. The integration of serological screening with molecular approaches, particularly proviral load quantification, may improve the identification of animals with greater transmission potential and provide a more robust basis for strategic BLV control and management programs aimed at reducing viral dissemination and its long‐term sanitary and productive impacts.

**Nomenclature**
BLV: Bovine leukemia virus
MY: Milk yield
SCC: Somatic cell count
SC: Services per conception
CI: Calving interval
WOAH: World Organization for Animal Health
95% CI: Confidence interval
RR: Relative risk
OR: Odds ratio

## Author Contributions

Conceptualization: Karla Cristina Resplandes da Costa Paz and Cecília Nunes Moreira. Data curation: Karla Cristina Resplandes da Costa Paz. Formal analysis: Karla Cristina Resplandes da Costa Paz, Ísis Assis Braga, Eduardo Vignoto Fernandes, Adeliane Castro da Costa, and Cecília Nunes Moreira. Funding acquisition: Cecília Nunes Moreira. Investigation: Karla Cristina Resplandes da Costa Paz. Supervision: Cecília Nunes Moreira and Ísis Assis Braga. Methodology: Karla Cristina Resplandes da Costa Paz, Leonardo Costa Paes, Dirceu Guilherme de Souza Ramos, Rosangela Maria Rodrigues, Eduardo Vignoto Fernandes, João Batista Alves de Souza, Adeliane Castro da Costa, Ísis Assis Braga, and Cecília Nunes Moreira. Project administration: Cecília Nunes Moreira. Resources: Cecília Nunes Moreira. Validation: Cecília Nunes Moreira and Ísis Assis Braga. Visualization: all authors. Writing–original draft: Karla Cristina Resplandes da Costa Paz, Ísis Assis Braga, and Cecília Nunes Moreira. Writing–review and editing: all authors.

## Funding

We gratefully acknowledge the Coordenação de Aperfeiçoamento de Pessoal de Nível Superior (CAPES) for supporting the Wiley publication agreement and for the scholarships provided to Karla Cristina Resplandes da Costa Paz and Leonardo Costa Paes.

## Conflicts of Interest

The authors declare no conflicts of interest.

## Data Availability

The data that support the findings of this study are available from the corresponding author upon reasonable request.
